# Patterned vascularization in a directional ice‐templated scaffold of decellularized matrix

**DOI:** 10.1002/elsc.202100034

**Published:** 2021-07-01

**Authors:** Li Shen, Xiuyue Song, Yalan Xu, Runhua Tian, Yin Wang, Peifeng Li, Jing Li, Hao Bai, Hai Zhu, Dong Wang

**Affiliations:** ^1^ Institute for Translational Medicine The Affiliated Hospital of Qingdao University Medical College Qingdao University Qingdao P. R. China; ^2^ School of Basic Medicine Qingdao University Qingdao P. R. China; ^3^ Department of Clinical Laboratory The Affiliated Hospital of Qingdao University Qingdao P. R. China; ^4^ State Key Laboratory of Chemical Engineering College of Chemical and Biological Engineering Zhejiang University Hangzhou P. R. China; ^5^ Department of Urology Qingdao Municipal Hospital Affiliated to Qingdao University Qingdao P. R. China

**Keywords:** decellularized extracellular matrix, directional ice templating, microvessels, tissue engineering, vascularization

## Abstract

Vascularization is fundamental for large‐scale tissue engineering. Most of the current vascularization strategies including microfluidics and three‐dimensional (3D) printing aim to precisely fabricate microchannels for individual microvessels. However, few studies have examined the remodeling capacity of the microvessels in the engineered constructs, which is important for transplantation in vivo. Here we present a method for patterning microvessels in a directional ice‐templated scaffold of decellularized porcine kidney extracellular matrix. The aligned microchannels made by directional ice templating allowed for fast and efficient cell seeding. The pure decellularized matrix without any fixatives or cross‐linkers maximized the potential of tissue remodeling. Dramatical microvascular remodeling happened in the scaffold in 2 weeks, from small primary microvessel segments to long patterned microvessels. The majority of the microvessels were aligned in parallel and interconnected with each other to form a network. This method is compatible with other engineering techniques, such as microfluidics and 3D printing, and multiple cell types can be co‐cultured to make complex vascularized tissue and organ models.

**Abbreviation**
ECM: extracellular matrix

## 1 INTRODUCTION

Vascularization is fundamental for engineering artificial organs, as microvessels are required to transport nutrients and oxygen to the cells inside large‐scale tissues [1]. Various strategies have been developed to culture microvessels in vitro [2, 3]. One common method is to seed vascular endothelial cells in a three‐dimensional (3D) hydrogel [2, 4, 5]. The endothelial cells used in these studies are usually cell lines, human umbilical vein endothelial cells, or induced pluripotent stem cell‐derived endothelial cells [2, 6]. Collagen, Matrigel, fibrin, and decellularized extracellular matrix (ECM) from animal organs have been used in these 3D hydrogel systems [2]. In recent years, decellularized ECM from porcine organs has been widely used in tissue engineering and a first‐in‐man clinical trial has proved its safety and feasibility [7]. Decellularized ECM is mainly composed of collagen and can be digested by pepsin to make a hydrogel, which can be injected in vivo for tissue regeneration or processed in vitro for tissue engineering [8, 9, 10]. More recently, microfluidics has been used to build microchannels, which are seeded with endothelial cells, to mimic microvascular structure [6, 8, 11, 12, 13, 14, 15]. The endothelial cells can also be mixed in a 3D hydrogel and self‐assemble to form a microvascular network within the microfluidic channels [16, 17]. However, these techniques can only generate microvascular models at the scale of hundreds of micrometers, which is not suitable for large‐scale tissue engineering.

**PRACTICAL APPLICATION**

Vascularization is a challenge in the field of tissue engineering. Currently, most methods aim to precisely fabricate microchannels for individual microvessels. Few studies have examined the remodeling capacity of the microvessels in the engineered constructs. Here, we present a model platform for engineering aligned microvessels in a directional ice‐templated scaffold of decellularized porcine kidney extracellular matrix. The patterned microchannels fabricated by ice templating allowed for fast and efficient cell seeding. The pure decellularized matrix without any fixatives or cross‐linkers maximized the potential of vascular remodeling. Dramatical microvascular remodeling happened in 2 weeks of culture, from small primary microvessel segments to a well‐developed aligned microvascular network. This model platform is compatible with other engineering techniques and multiple cell types can be co‐cultured to make complex vascularized tissue and organ models.

To fabricate large‐scale vascularized tissues at the scale of millimeters or centimeters, 3D printing techniques have been developed over the past few years [18, 19]. In this approach, a sacrificial material is first printed, which is embedded in another type of biomaterial. Microchannels are created by dissolving the sacrificial material and endothelial cells are then seeded into the microchannels, where they adhere to channel walls and form tubular structures [18, 19]. The endothelial cells can also be mixed in a hydrogel bioink, which is then 3D‐printed to create a tubular microvascular structure [20]. In the 3D printed microvascular grafts, the vascular microchannels are precisely designed for individual microvessels [21]. However, few studies have examined the remodeling capacity of these engineered microvessels, which is an important issue for transplantation in vivo.

Directional ice templating, also called freeze casting, is another powerful technique for producing large‐scale microporous scaffolds [22, 23]. It generates aligned ice crystals, which are then freeze‐dried to make an aligned 3D porous structure [22, 23]. This simple and versatile technique has been applied to various materials, including inorganic materials [24, 25], nanoparticles [22], and synthetic polymers [22, 26]. At present, however, only a few natural biological materials have been investigated by directional ice templating, including chitosan, gelatin, and collagen [19, 27‐29]. Because collagen and its hydrolyzed product gelatin have weak mechanical properties, fixatives such as glutaraldehyde [28] and sodium polyphosphate [19, 29] were usually added to enhance their mechanical strength, which may prevent the scaffold from being remodeled by the cells. To date, few studies have reported the fabrication of ice‐templated scaffolds from pure collagen or decellularized ECM.

In this study, we presented a method to fabricate large‐scale patterned vascularized tissues using pure decellularized porcine kidney ECM without any fixatives. A 3D porous scaffold was fabricated from decellularized ECM by directional ice templating. In the proof‐of‐concept study, primary microvessels grew well in the scaffold and self‐assembled into a well‐developed vascular network through an active remodeling process.

## 2 MATERIALS AND METHODS

### 2.1 Preparation of decellularized porcine kidney ECM hydrogel

The decellularization procedure followed the previous protocols with minor modifications [8, 9, 30]. The kidney of 1‐year old porcine was purchased from the market. All the following procedures were performed in an aseptic environment. The kidney cortex was harvested and sliced into 2 mm thick pieces. The small pieces of cortex tissue were first stirred in 1% SDS for 3 days and then in 1% Triton X‐100 for 1 day. The decellularized tissue was washed in distilled water for 5 days before digestion in 1 mg/mL pepsin in 0.01 N HCl. The digested decellularized ECM hydrogel solution was frozen at ‐80°C for long‐term storage.

To test the gel formation performance, the ECM hydrogel was neutralized to pH = 7.0 by 1 M NaOH, buffered in 1 × PBS, incubated at 37°C for 1 h.

### 2.2 Characterization of decellularized ECM

To characterize the quality of decellularization, the tissues before and after decellularization were fixed in 4% PFA for 30 min, immersed in 15% and then 30% sucrose solution, and embedded in OCT for cryosection. H&E staining was performed according to the manufacture protocol (Solarbio, Cat#G1121).

SDS‐PAGE was performed to qualitatively examine ECM protein content. Briefly, a small piece of native porcine kidney tissue was lysed in RIPA buffer. Digested ECM hydrogel was used because the decellularized ECM did not dissolve in common lysis buffers. The protein concentration of each sample was determined by BCA assay. Proteins were separated by 10% SDS‐PAGE, stained by Coomassie Blue, digested by trypsin and identified by LC‐MS/MS as previously described [31].

DNA quantification was performed by PicoGreen assay following previous reports [10]. Freeze‐dried porcine kidney tissues before and after decellularization were digested in Proteinase K overnight. DNA was quantified by the PicoGreen dsDNA Kit (Yeasen, Cat#12641ES01). Sulfated glycosaminoglycan (GAG) was quantified by the DMMB assay following the previous report [10].

### 2.3 Fabrication of directional ice‐templated ECM scaffold

The decellularized ECM hydrogel was adjusted to pH = 7.0 by 1 M NaOH solution before ice templating. To induce directional ice crystal formation, the neutralized ECM hydrogel solution was added into a silicone chamber of 3‐mm width, 10‐mm length, and 20‐mm height, on the top of a copper plate, which was placed on a liquid nitrogen container. To induce random ice crystal formation, the silicone chamber was made on a piece of silicone and put into a ‐80°C freezer. The frozen samples were freeze‐dried and used for experiments. For the cell seeding test, a few drops of mCherry‐231 cells of 10^7^/mL were added to the distal end of the ECM scaffold and imaged by confocal microscopy.

For SEM examination, the frozen samples were cut into several pieces to expose the internal cross‐sectional or longitudinal structure before freeze‐drying. For quantification of microchannel width, at least four images with nine microchannels per image were measured for each sample.

### 2.4 Animal experiments

All the animal experiments followed the Ministry of Science and Technology guide for laboratory animal care and use and approved by the animal care and use committee of Qingdao University. Male SD rats of 8–10 weeks were euthanized by an overdose of isoflurane until they ceased breathing and followed by bilateral thoracotomy. Dorsal hair was shaved and disinfected with 75% ethanol before the harvest of subcutaneous soft connective tissue.

### 2.5 Primary cell isolation and culture

The subcutaneous soft connective tissue was digested in 2 mg/mL collagenase type 1 (Wathington, Cat#LS004196) and 2 mg/mL dispase (Wathington, Cat#LS02109) in DMEM (Invitrogen, Cat#12430) in a 37°C water bath. The cell suspension was filtered through a 30 μm strainer and microvessels were collected from the strainer mesh. The primary microvessels were cultured in the endothelial growth medium. About 6 × 10^4^ microvessel segments were seeded to the ECM scaffolds with the size of 3 mm × 10 mm × 20 mm.

### 2.6 Immunostaining

The cells and tissues were fixed in 4% PFA for 30 min at room temperature, washed with PBS, treated with 0.1% Triton X‐100 for 10 min, blocked in 5% normal donkey serum, incubated in the following primary antibodies: Collagen I (Proteintech, Cat#14695‐1‐AP), Collagen IV (Proteintech, Cat# 55131‐1‐AP), CD31 (Abcam, Cat#ab28364), VE‐Cadherin (Santa Cruz, Cat#sc‐52751), vWF (Protentech, Cat#11778‐1‐AP). After 2 h of incubation in primary antibodies, the cells were washed in PBS 3 times and incubated in secondary antibodies (Invitrogen, Cat# A10040, A10036) for 2 h. Cell nuclei were stained by DAPI. Images were taken by a Leica SP8 confocal microscope.

## 3 RESULTS AND DISCUSSION

### 3.1 Decellularization of porcine kidney cortex

**FIGURE 1:**
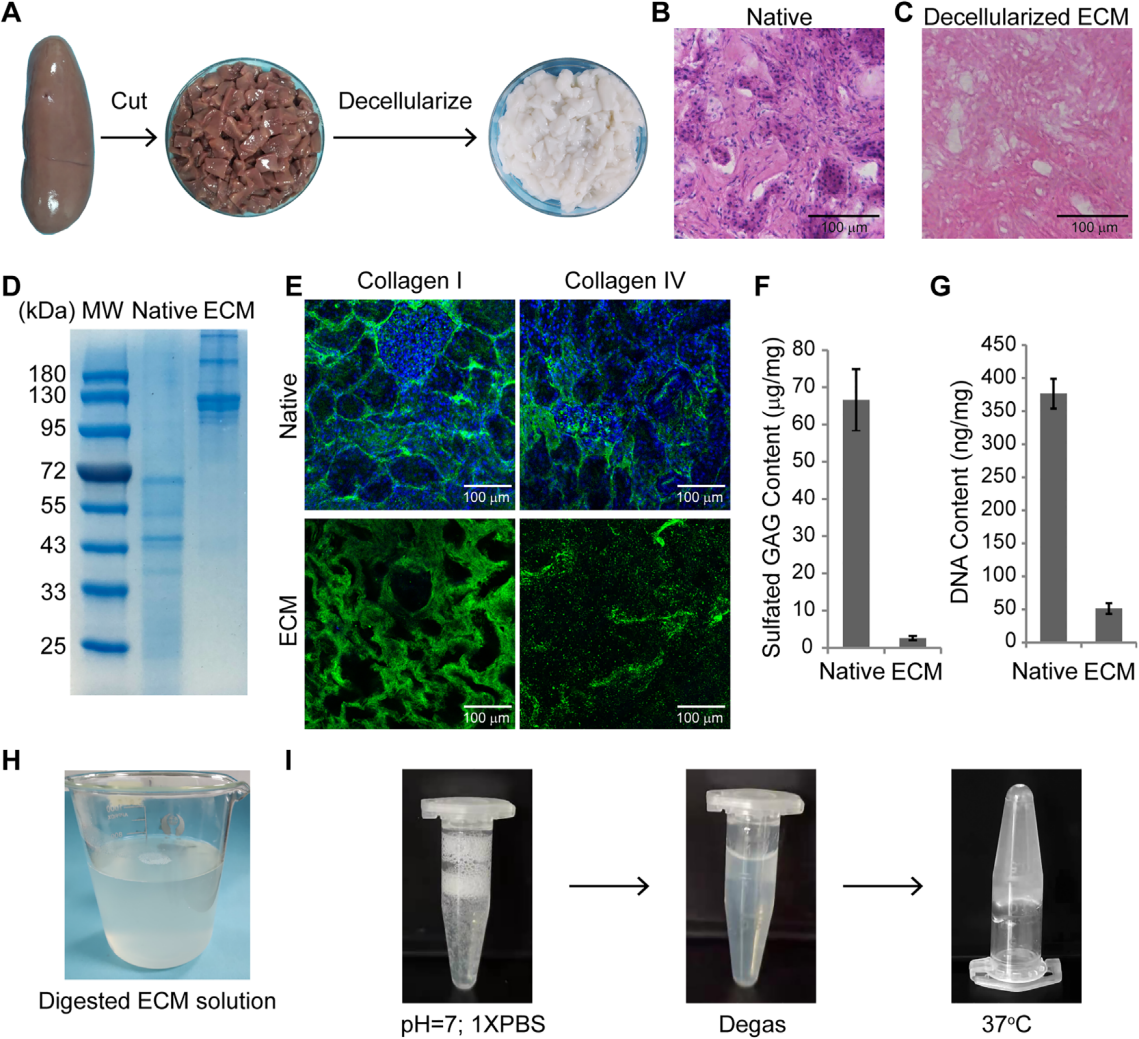
Preparation of decellularized porcine kidney ECM hydrogel. (A) Porcine kidney cortex was cut into small pieces of 2 mm width, decellularized in 1% SDS and 1% TritonX‐100. (B and C) H&E staining of the kidney tissue section before (B) (Native) and after (C) decellularization (decellularized ECM). (D) SDS‐PAGE gel. (E) Immunostaining of the porcine kidney tissue before and after decellularization by the antibodies against Collagen I and IV. (F) Quantification of sulfated GAG content. (G) Quantification of DNA content. The data were presented as mean +/‐ SD. H, The decellularized ECM was digested in 1 mg/mL pepsin in 0.01 N HCl to produce a hydrogel solution. (I) The ECM hydrogel was neutralized by 1 M NaOH, buffered in 1 × PBS, degassed by centrifuge at 500 rpm and 4°C for 10 min, incubated at 37°C for 1 h, and finally formed a gel. Scale bars, 100 μm

In this study, the porcine kidney cortex was decellularized in SDS and Triton following previous reports [8‐10, 30]. The decellularized kidney ECM had a white appearance (Figure 1A). The majority of the cells were removed as evidenced by hematoxylin and eosin staining (Figure 1B and 1C) and DNA quantification (Figure 1G). The major component of the decellularized kidney ECM was collagen I, which was resolved by SDS‐PAGE (Figure 1D), identified by LC‐MS/MS analysis of the excised gel bands (data not shown) and confirmed by immunostaining (Figure 1E). There were smaller amounts of collagen IV and sulfated GAG left in the decellularized ECM compared to previous reports (Figure 1E and 1F) [10]. The decellularized ECM was digested in pepsin/HCl to make an ECM hydrogel solution (Figure 1H). The ECM hydrogel exhibited efficient gel formation when it was neutralized to pH = 7.0 by 1 M NaOH and buffered in 1×PBS solution (Figure 1I). There were bubbles during the neutralization process, which can be eliminated by centrifugation at 500 rpm and 4°C for 10 min (Figure 1I). We diluted the ECM solution and found that it could form a gel at 12, 6, 3, and 1.5 mg/mL.

The amount of Collagen IV and sulfated GAG of decellularized ECM in this study are lower than those in previous report [10], possibly due to the harsh decellularization conditions in our study. However, the DNA content of decellularized ECM in our study is higher than those of previous reports [10], although this did not affect the gelation property and cell culture (see following Section 3.4 and Figure  6). One possible reason is that the age of kidney donor is older (1 year) that those in other reports (3‐4 months) [10].

### 3.2 Patterned porous ECM scaffold by directional ice templating for efficient cell seeding

**FIGURE 2:**
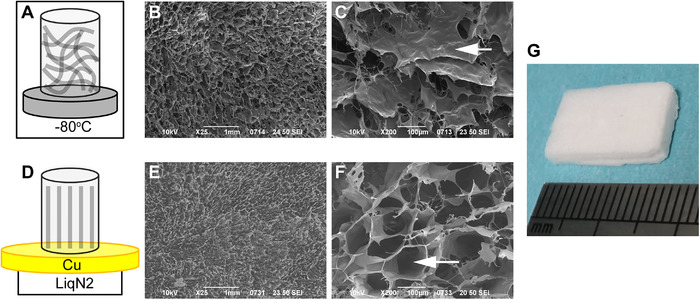
SEM characterization of the directional ice‐templated ECM scaffold. The ECM (6 mg/mL) was processed by the random (A) and directional (D) ice templating. The cross‐sections of the scaffolds of random (B and C) and directional (E and F) ice‐templating groups were imaged by SEM. (G) Photograph of the directional ice‐templated ECM scaffold. The scale bars in B and E, 1 mm. The scale bars in C and F, 100 μm

In our previous work, we fabricated a directional ice‐templated hydroxyapatite scaffold for efficient cell seeding [24]. In this study, we made a simplified ice‐templating setup (Figure 2). In the control (random freezing) group, bulk ECM solution was frozen in a ‐80°C freezer (Figure 2A). In the directional ice‐templating group, the ECM chamber was fixed on a copper plate, which was placed on a liquid nitrogen container; consequently, the ECM was frozen from the bottom to top direction (Figure 2D). By adjusting the distance between liquid nitrogen and the copper plate, we can freeze the ECM hydrogel in a slow (about 1 mm/min) or fast (about 3.3 mm/min) mode. We first examined the slow freezing group and characterized the morphology and cell seeding efficiency of the directional ice‐templated ECM scaffolds.

Instead of the random fibers made by conventional methods [10], the ice‐templating method in this study produced microsheets of ECM (Figure 2). In the random freezing group, the cross‐sectional surface of the scaffold consisted of microsheets of ECM, which were stacked together layer by layer, leaving few micropores (Figure 2B and 2C). By contrast, directional ice‐templated ECM had microchannels that were aligned in parallel with the freezing direction (Figure 2E and 2F). The size of the directional ice‐templated ECM scaffold can be at the scale of centimeters (Figure 2G), which makes it suitable for engineering large‐scale tissues.

**FIGURE 3:**
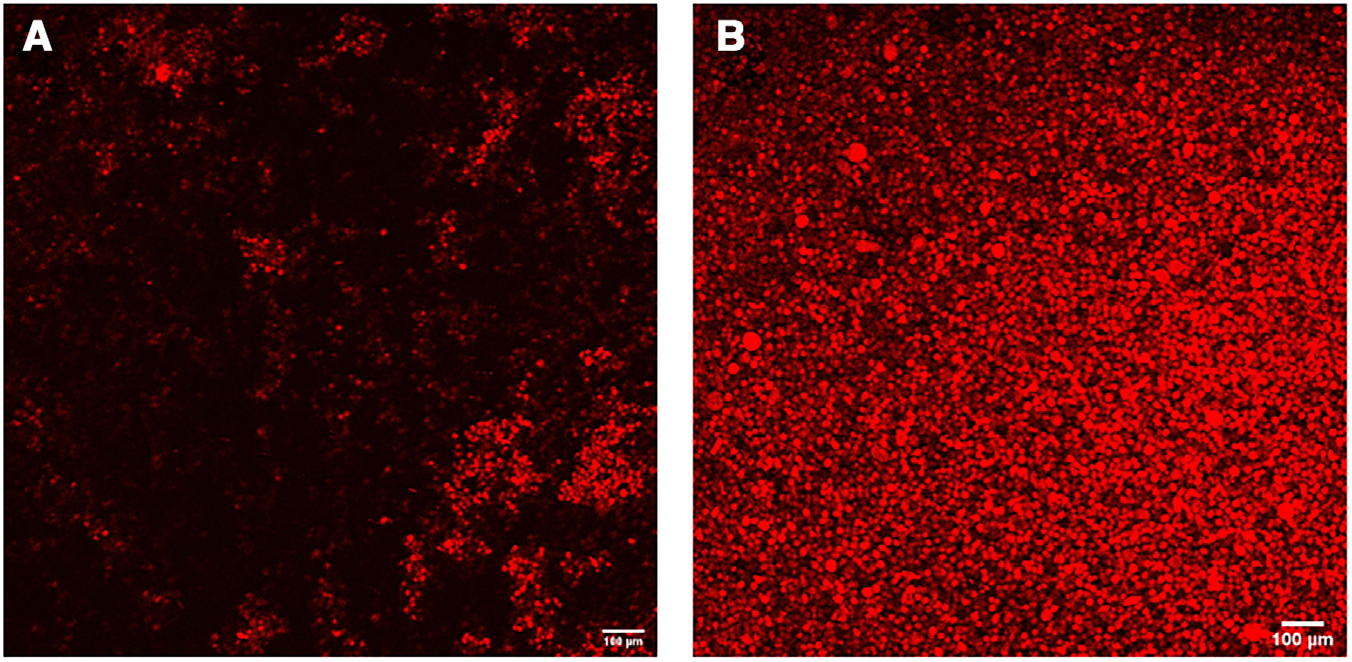
Cell seeding test. The random (A) and directional ice‐templated (B) scaffolds were seeded with mCherry‐231 cells and imaged by confocal microscopy

To test the cell seeding efficiency, we added drops of mCherry‐231 cells to the distal ends of the scaffolds. The cells can hardly get inside the random ECM scaffold and only formed local aggregates in the peripheral region (Figure 3A). In the directional ice‐templated scaffold, the cells quickly get through the microchannels with uniform distribution inside the scaffold (Figure 3B). This is consistent with our previous work in the directional ice‐templated hydroxyapatite scaffold [24].

### 3.3 Microchannel morphology modulated by ECM concentration and freezing speed

**FIGURE 4:**
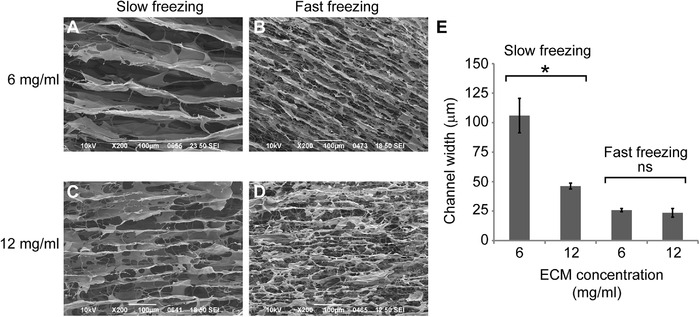
Microchannel size modulated by ECM concentration and freezing speed. The Longitudinal sections of directional ice‐templated ECM scaffolds of 6 mg/mL (A and B) and 12 mg/mL (C and D) at different freezing speeds were imaged by SEM. (E) quantification of the microchannel width of different groups. Student's *t*‐test was performed to analyze significant differences between groups. *, *P* < 0.01. ns, not significant. The data were presented as mean +/‐ SD. Error bars represent standard deviation. The sample sizes for slow freezing 6 and 12, fast freezing 6 and 12 mg/ mL groups: *n* = 4, 8, 7, and 4 respectively. Scale bars, 100 μm

In previous reports, multiple materials with different molecular weights have been investigated by directional ice‐templating, such as hydroxyapatite (< 1 kDa), poly vinyl alcohol (9 kDa), polycaprolactone (10 kDa), and gelatin (40‐50 kDa) [22, 24, 25, 28, 32]. The microstructure of directional ice‐templated scaffolds based on these materials has been well illustrated to be regulated by freezing speed and solute concentration [22, 24, 25, 28, 32]. In this study, the ECM solution is mainly composed of collagen, which has a larger molecular weight (> 100 kDa) (Figure 1D) and a longer molecular structure [33]. We observed the similar trends to previous reports and some different aspects in our experiments (Figure 4A‐E).

Two concentrations (6 and 12 mg/mL) of ECM and two freezing speeds (1 and 3.3 mm/min) were examined in this study. Slower freezing speed gave rise to wider microchannels for both concentrations of ECM (Figure 4). In the slow freezing group, the microchannel widths were around 100 μm for 6 mg/mL, and 50 μm for 12 mg/mL ECM respectively (Figure 4A, 4C and 4E). However, the microchannel widths had no significant difference between the two concentrations at the fast freezing speed (Figure 4E). On the other hand, we noticed small defects in the microchannel walls of the 6 mg/mL ECM scaffold (Figure 4A). In the scaffold of 12 mg/mL ECM, the defects were much more obvious and the channel walls were discontinuous in some local regions (Figure 4C). In the fast freezing group, the ECM morphology was like fibers (Figure 4B and 4D), rather than the microsheets observed in the slow freezing group (Figure 4A and 4C).

The morphological changes of ECM microchannel walls under different freezing speeds may reflect the distinct molecular properties of ECM, which is mainly composed of collagen I in this study. Collagen has a larger molecular weight and a longer structure compared to the materials in previous reports [22, 24, 25, 28, 32]. Slower freezing speed may make it possible for collagen molecules to have enough time to form a network during freeze casting and thus produces microsheets with a larger area. By contrast, faster freezing speed can only make collagen bundles and produces fiber‐like structures, which is commonly observed in conventional studies [10]. This phenomenon can be utilized in tissue engineering depending on the specific requirements. The directional ice‐templated ECM scaffold at conditions of 6 mg/mL and slow freezing speed had a proper channel size (around 100 μm) for microvascular engineering. The micropores in the channel walls may allow for vascular branching and network formation.

### 3.4 Microvascular remodeling and patterning in the directional ice‐templated ECM scaffold

**FIGURE 5:**
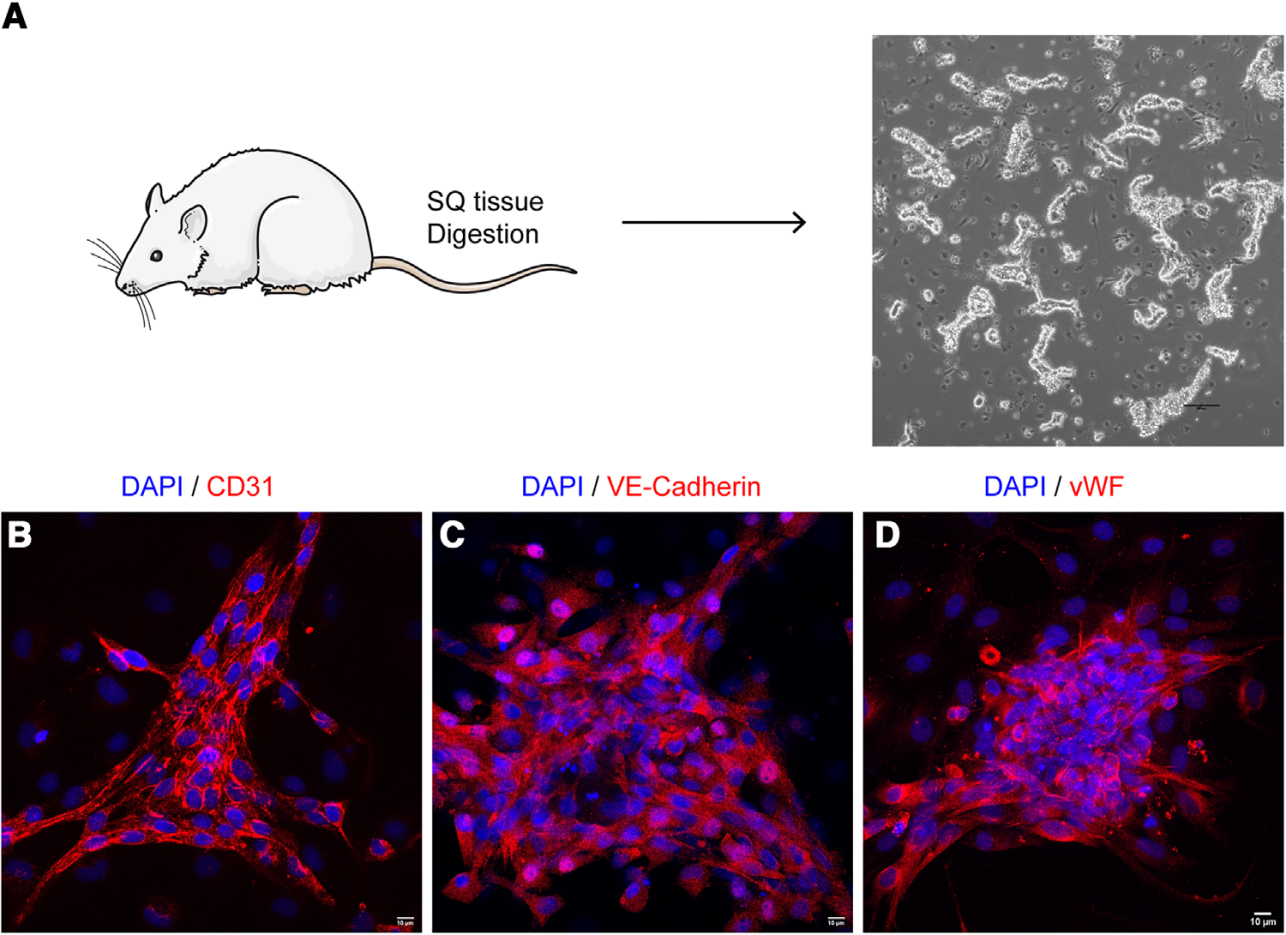
Microvessel culture in vitro. (A) Subcutaneous (SQ) tissue was harvested from SD rats and digested to segments of microvessels, which were imaged by phase‐contrast microscopy. (B‐D) The microvessels cultured in vitro were immunostained by the antibodies against CD31 (B), VE‐Cadherin (C), vWF (D) and imaged by confocal microscopy. Cell nuclei were stained by DAPI. The scale bar in A, 100 μm. The scale bars in B‐D, 10 μm

**FIGURE 6:**
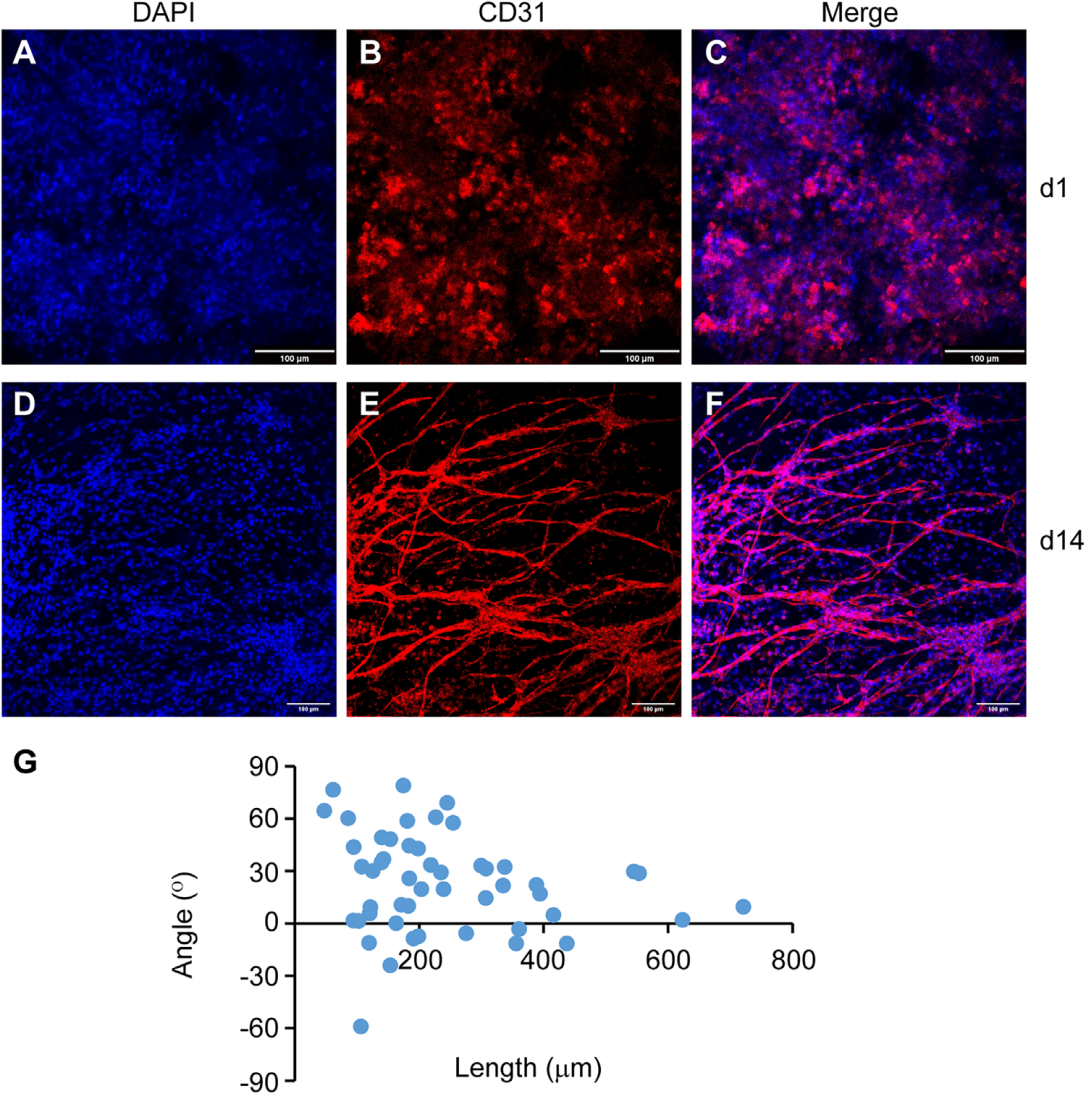
Patterned microvessels in the directional ice‐templated ECM scaffolds. The scaffolds loaded with microvascular cells were cultured in vitro for 1 (A‐C) and 14 (D‐F) days, and immunostained by the antibody against CD31. (G) Quantification of the lengths and angles of the microvessels (D‐F) at day 14. Scale bars, 100 μm

In contrast to most studies that use cell lines or primary human umbilical vein endothelial cells, we isolated primary microvascular cells from rat subcutaneous soft connective tissue (Figure 5A). After enzymatic digestion, the primary cells were filtered through a 30 μm strainer, and larger segments of microvessels were harvested for experiments (Figure 5A). Microvascular endothelial cells expanded in vitro expressed endothelial‐specific markers such as CD31, VE‐Cadherin, and vWF (Figure 5B‐D). The primary microvessels were seeded into the directional ice‐templated ECM scaffolds, which were made of 6 mg/mL ECM by the slow freezing method with a channel width of around 100 μm (Figure 4A). During in vitro culture for 2 weeks, the microvessels grew dramatically and self‐assembled to form long microvessels aligned in parallel inside the scaffold (Figure 6). The microvessels were further interconnected with each other to form a network (Figure 6D‐G), which may be facilitated by the defects in the microchannel walls (Figure 4). The use of pure ECM without fixatives or cross‐linkers in the scaffold may also facilitate microvascular remodeling.

Many tissue engineering studies use endothelial cell lines, HUVECs or the endothelial cells derived from induced pluripotent stem cells [2]. The advantage of these cells is their easy availability and can be obtained in a large amount. However, they may have potential problems of immune response, tumor formation, and other unknown risks in clinical settings. At present, primary autologous endothelial cells isolated from adult tissue are the most suitable cell source for clinical application. Our study presented a platform for integrating primary autologous endothelial cells and directional ice‐templated ECM scaffold, which can be further improved by the combination with other techniques, such as 3D printing, and promote the development of tissue vascularization.

## 4 CONCLUDING REMARKS

Patterned vascularization is fundamental for engineering large‐scale tissues and will pave the way for fabricating artificial organs. In this proof‐of‐concept study, we presented a method for patterning large‐scale vasculature in a porous scaffold of decellularized porcine kidney ECM. The patterned microchannels were fabricated by directional ice templating and the microchannel morphology can be modulated by ECM concentration and freezing speed. The aligned microchannels allowed for fast and efficient cell seeding. The defects in the channel walls allow for vascular branching and network formation. Primary microvessels grew well in the scaffold and formed an aligned microvascular network through an active remodeling process. We believe that the use of pure decellularized ECM, without any fixatives or cross‐linkers, is critical for microvascular remodeling and patterning. The scaffold can be made at the scale of centimeters, enabling the fabrication of large‐scale vascularized tissues.

In this study, the cells were cultured in static culture plates. Further studies should investigate the growth of microvessels in dynamic culture conditions, for example, in the presence of fluid flow. The mechanical properties of the directional ice‐templated ECM scaffold and vascularized tissues need to be characterized in the future. Given that angiogenesis is a complex process involving multiple types of cells, it would be of special interest to investigate co‐culture systems of microvessels in combination with other types of cells, such as pericytes, stem cells, cardiac cells, or epithelial cells, for constructing functional vascularized tissues and organs.

## CONFLICT OF INTEREST

The authors have declared no conflicts of interest.

## Data Availability

The data that support the findings of this study are available from the corresponding author upon reasonable request.
